# Depletion of nuclear import protein karyopherin alpha 7 (KPNA7) induces mitotic defects and deformation of nuclei in cancer cells

**DOI:** 10.1186/s12885-018-4261-5

**Published:** 2018-03-27

**Authors:** Elisa M. Vuorinen, Nina K. Rajala, Teemu O. Ihalainen, Anne Kallioniemi

**Affiliations:** 1 BioMediTech Institute and Faculty of Medicine and Life Sciences, University of Tampere, PL 100, 33014 Tampere, Finland; 2 BioMediTech Institute and Faculty of Biomedical Sciences and Engineering, Tampere University of Technology, University of Tampere, PL 100, 33014 Tampere, Finland; 3 Tampere Imaging Facility, BioMediTech Institute and Faculty of Medicine and Life Sciences, University of Tampere, PL 100, 33014 Tampere, Finland; 4 Fimlab Laboratories, Biokatu 4, 33520 Tampere, Finland

**Keywords:** KPNA7, Importin alpha 8, Nuclear transfer, Nuclear morphology, Cell proliferation, Mitosis

## Abstract

**Background:**

Nucleocytoplasmic transport is a tightly regulated process carried out by specific transport machinery, the defects of which may lead to a number of diseases including cancer. Karyopherin alpha 7 (KPNA7), the newest member of the karyopherin alpha nuclear importer family, is expressed at a high level during embryogenesis, reduced to very low or absent levels in most adult tissues but re-expressed in cancer cells.

**Methods:**

We used siRNA-based knock-down of KPNA7 in cancer cell lines, followed by functional assays (proliferation and cell cycle) and immunofluorescent stainings to determine the role of KPNA7 in regulation of cancer cell growth, proper mitosis and nuclear morphology.

**Results:**

In the present study, we show that the silencing of KPNA7 results in a dramatic reduction in pancreatic and breast cancer cell growth, irrespective of the endogenous KPNA7 expression level. This growth inhibition is accompanied by a decrease in the fraction of S-phase cells as well as aberrant number of centrosomes and severe distortion of the mitotic spindles. In addition, KPNA7 depletion leads to reorganization of lamin A/C and B1, the main nuclear lamina proteins, and drastic alterations in nuclear morphology with lobulated and elongated nuclei.

**Conclusions:**

Taken together, our data provide new important evidence on the contribution of KPNA7 to the regulation of cancer cell growth and the maintenance of nuclear envelope environment, and thus deepens our understanding on the impact of nuclear transfer proteins in cancer pathogenesis.

**Electronic supplementary material:**

The online version of this article (10.1186/s12885-018-4261-5) contains supplementary material, which is available to authorized users.

## Background

Eukaryotic cells are compartmentalized to contain distinct organelles such as the nucleus. The separation of the nucleus from the cytoplasm by the nuclear envelope forms a barrier across which large macromolecules, such as proteins, need to be transported. This allows the physical separation of transcription events in the nucleus and protein translation in the cytoplasm, thereby providing the cell an additional system for the regulation of protein function [1, 2]. The nucleocytoplasmic transport of proteins is carried out by specific transport machinery where proteins are shuttled in and out of the nucleus through the nuclear pore complexes (NPC) at the nuclear envelope [3]. In the classical protein import, karyopherin alpha (KPNA, also known as importin alpha) recognizes the protein containing a nuclear localization signal (NLS) [4–6]. The KPNA-cargo complex then binds to karyopherin beta 1 (KPNB1, also known as importin beta) that docks to the NPC and mediates the transport to the nucleus, where the cargo is released when the GTP-binding nuclear protein Ran (Ran-GTP) binds to KPNB1 [6, 7].

Defects in nuclear import, for instance due to abnormal function of members of the nuclear transport machinery, result in incorrect localization of proteins that might subsequently lead to a diversity of diseases, including cancer [1, 8–12]. For example, the tumor suppressor protein p53 has been shown to be inactivated in cancer due to a truncated form of KPNA that was incapable of transporting p53 into the nucleus, its proper location of action [13, 14]. Currently, the strongest evidence on the role of karyopherins in cancer pathogenesis comes from studies on KPNA2. KPNA2 is upregulated in a large variety of tumor types and its elevated expression is associated with an increased degree of malignancy, tumor spread and poor patient outcome [15, 16]. KPNA2 overexpression is already present in early lesions indicating that it is not merely a marker of advanced disease but actively participates in the pathogenesis process [15]. This notion is supported by functional studies where enhanced KPNA2 expression leads to increased cell proliferation and migration [15]. Other KPNAs have also been implicated in cancer. For example, KPNA4 was recently shown to promote the migration and metastatic potential of prostate cancer cells [17]. These data illustrate that alterations in nuclear transport are important players in cancer pathogenesis.

The karyopherins in the cells are vital to proper nuclear transport but their functional roles are even more diverse. They participate in the assembly of the mitotic spindle where the duplicated chromosomes are aligned during mitosis, and thereby ensure the fidelity of cell division [18, 19]. Karyopherins bind the spindle assembly factors (SAFs), keeping them inactive, and release them in an appropriate location near mitotic chromosomes thus preventing mislocalization of the spindle [19]. The release of SAFs is regulated by the differential concentration of Ran-GTP between the nucleus and cytoplasm, which is maintained by guanine nucleotide exchange factors (GEFs) in the nucleus and GTPase activating proteins (GAPs) in the cytoplasm [20]. The Ran-GTP gradient is also preserved around the chromatin after the dissociation of the nuclear envelope during mitosis [20]. Subsequent to chromosome separation, karyopherins are also involved in the reassembly of the nuclear envelope that consists of the inner and outer nuclear membranes and associated proteins, mainly lamins and the NPC proteins [18, 19, 21].

The human karyopherin alpha family consists of seven highly conserved members, with KPNA7 being the most recently identified, divergent and least studied member [22, 23]. *KPNA7* is mainly expressed during early embryogenesis and in oocytes in different animals [24–26] and has been identified as one of the target genes for the 7q21-22 amplicon in pancreatic cancer [27]. However, the precise function of KPNA7 in human cells remains elusive. In our previous work we pinpointed KPNA7 as a regulator of malignant properties in pancreatic cancer cells with high KPNA7 expression [28]. Here we extend these findings to show that even low KPNA7 expression plays an important role in the proliferation of both pancreatic and breast cancer cells. Furthermore, our data demonstrate that KPNA7 has a key role in the proper formation of the mitotic spindle and in the maintenance of nuclear morphology.

## Methods

### Cell lines

Hs700T, MIA PaCa-2 and SU.86.86 pancreatic cancer cell lines; MCF-7, T-47D, MDA-MB-231 and MDA-MB-453 breast cancer cell lines and hTERT-HPNE normal pancreas epithelial cell line were purchased from the American Type Culture Collection (ATCC, Manassas, VA, USA). The cell lines were authenticated by genotyping and were grown under recommended culture conditions. The cells were regularly tested for Mycoplasma infection.

### Gene expression analysis

Total RNA was extracted using RNeasy Mini kit (Qiagen, Hilden, Germany). Quantitative real-time PCR (qRT-PCR) was performed using the Roche LightCycler 2.0 instrument (Roche, Mannheim, Germany) with LightCycler® TaqMan® Master reaction mix (Roche). Universal Probe Library (Roche) probes and associated primers (Sigma Aldrich, St Louis, MO, USA) and Roche’s Reference Gene Assay for HPRT was utilized for normalization. All primer and probe sequences are listed in Additional file 1: Table S1.

### Gene silencing

Four specific small interfering RNAs (siRNAs) against the *KPNA7* gene were designed using the siRNA Selection Program of the Whitehead Institute, Cambridge, MA, USA and the siRNAs were obtained from Dharmacon (Lafayette, CO, USA). A pool containing an equal concentration of each of the four siRNAs was prepared. SMAD5 SMARTpool siRNA was acquired from Dharmacon. An siRNA targeting the firefly luciferase (LUC) gene (Sigma Aldrich) was used as a control in all transfections. Transfections were performed either on 24-well or 6-well plates using 10 nM siRNA and Interferin reagent (Polyplus-Transfection, San Marcos, CA, USA) as described previously [27]. The efficacy of the gene silencing was verified in each experiment using qRT-PCR. The number of cells plated per experiment are listed in Additional file 1: Table S2.

### Cell growth and cell cycle analysis

For cell proliferation assays, the cells were seeded (for cell numbers see Additional file 1: Table S2) on a 24-well plate and transfected with KPNA7 or LUC siRNAs as described above. The cells were counted at 72 h or 96 h after transfection using a Coulter Z2 Coulter Counter (Beckman Coulter, San Diego, CA, USA). In cell cycle studies, the cells were seeded (for cell numbers see Additional file 1: Table S2) on 6-well plates, transfected with KPNA7 or LUC siRNAs and analyzed 96 h after transfection. The cells were collected by trypsinization and suspended to 500 μL hypotonic staining buffer (0.1 mg/mL sodium citrate tribasic dehydrate, 0.03% Triton X-100, 50 μg/mL propidium iodide, 2 μg/mL RNase A) and the amount of propidium iodide incorporated was determined using flow cytometry (BD Accuri Cytometers, Ann Arbor, MI, USA). The cell cycle distributions were determined using the ModFit LT software (Verity Software House Inc., Topsham, ME, USA). All experiments were performed in six replicates and repeated at least twice.

### Immunofluorescence assays

Immunofluorescence (IF) was used to assess the localization of γ-tubulin, different nuclear envelope proteins and formation of stress fibers. The cells were plated on coverslips on 24-well plates (for cell number see Additional file 1: Table S2) and the IF stainings were performed as previously described [29]. The following antibodies and dilutions were used: anti-lamin A/C 1:200 (ab8984, Abcam, Cambridge, UK), anti-lamin B1 1:500 (ab16048, Abcam), anti-NUP153 1:1000 (ab24700, Abcam), anti-γ-tubulin 1:500 (ab179503, Abcam), anti-phospho Myosin Light Chain 2 1:200 (#3674S, Cell Signaling Technology, Danvers, MA, USA), Alexa Fluor 568 Phalloidin 1:200 (Molecular Probes, Eugene, OR, USA) and Alexa Fluor secondary antibodies 1:200 (Molecular Probes). The samples were mounted in ProLong Antifade Gold reagent with DAPI (Molecular probes). The fluorescently labeled cells were photographed using the Zeiss Apotome or the Zeiss LSM 780 laser scanning confocal microscope (Zeiss, Oberkoche, Germany).

### Quantification of the IF data

A total of 50 mitotic cells from γ-tubulin stained, siRNA treated cells were scored using the Zeiss Apotome (40X objective) and classified either as normal or abnormal based on the number of centrosomes and the structure of the mitotic spindle. The appearance of the nuclei was visually inspected from lamin A/C images obtained using the confocal microscope and a 40X objective. The nuclei were categorized as normal or aberrant and their number was determined from a minimum of six images. ImageJ software (US National Institute of Health, Bethesda, MD, USA) was used for the quantitation of the nuclear size and shape as well as nuclear aspects ratios in 50 randomly selected nuclei from samples stained with lamin A/C. In the case of NUP153 staining, ImageJ was used for the quantitation of the number of spots per nucleus.

### Western blotting

Cell lysates were separated in a 7% (for NUP153) or 10% (for lamins) SDS-PAGE gel. The proteins were transferred onto polyvinylidene fluoride (PVDF) membrane (Roche) using a tank blotter (NUP153 western) or Trans-Blot SD semi-dry transfer cell (Bio-Rad Laboratories, Hercules, CA, USA). The membrane was blocked with Blocking Reagent (Roche) in tris-buffered saline (TBS, 50 mM Tris-HCl, 150 mM NaCl, pH 7.5) for 1 h at RT. After blocking, the membrane was probed with primary antibody diluted in 3% BSA in 0.05% TBS-Tween-20 (TBST) overnight at 4 °C and subsequently with HRP-conjugated IgG secondary antibody (Vector Laboratories, Burlingame, CA, USA) 1:8000 in 0.05% TBST for 1 h at RT. The protein bands were detected with Pierce™ ECL Plus Western Blotting Substrate (ThermoFischer Scientific, Waltham, MA, USA). The following antibodies and dilutions were used: anti-lamin A/C 1:500 (ab2811, Abcam), anti-lamin B1 1:1000 (ab16048, Abcam), anti-NUP153 1:1000 (ab24700, Abcam) and anti-β-Tubulin 1:20,000 (T7816, Sigma-Aldrich).

### Statistical analyses

The Mann-Whitney test was used to statistically compare the means of the siKPNA7 and siLUC control groups.

## Results

### KPNA7 is essential for pancreatic and breast cancer cell proliferation

**Fig. 1:**
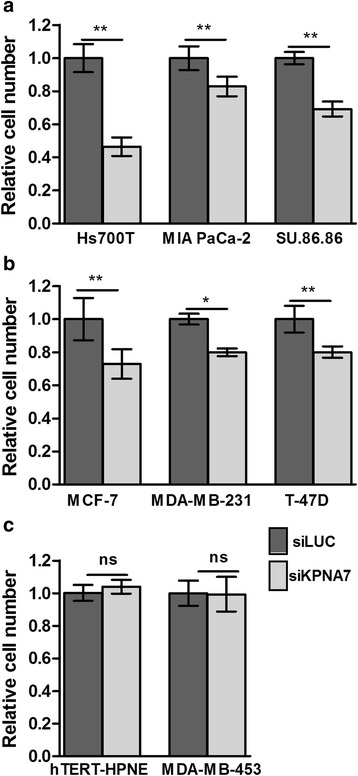
Silencing of KPNA7 inhibits cell proliferation in cells with endogenous *KPNA7* expression. *KPNA7* was silenced with siRNAs in (**a**) pancreatic and (**b**) breast cancer cell lines with different levels of endogenous *KPNA7* expression, as well as in (**c**) *KPNA7*-negative cell lines. A siRNA targeting the firefly luciferase gene (LUC) was used as a control. Mean cell numbers (± SD of six replicates) were determined 72 or 96 h post-transfection and normalized to the mean of siLUC control of each cell line. The silencing efficiencies were verified in each case with qRT-PCR and are shown in Additional file 1: Table S3. The experiments were repeated at least twice and data from one representative experiment is shown. *p < 0.05, **p < 0.01

We previously showed that amplification and subsequent overexpression of KPNA7 leads to promotion of cell growth in Hs700T and AsPC-1 pancreatic cancer cell lines [28]. To obtain a more comprehensive view on the functional role of this protein in cancer cells, we extended these studies to include pancreatic (MIA PaCa-2, SU.86.86) and breast (MCF-7, MDA-MB-231, T-47D) cancer cell lines without *KPNA7* amplification and with varying levels of endogenous KPNA7 expression (Additional file 2: Figure S1). KPNA7 was silenced in these cell lines as well as the Hs700T cells with siRNAs leading to a minimum of 80% decrease in mRNA levels (Additional file 1: Table S3). As previously shown [28], the silencing resulted in a dramatic reduction of cell growth in Hs700T cells as compared to controls transfected with siRNAs targeting the firefly luciferase (*LUC*) gene (Fig. 1a). Likewise, the other pancreatic and breast cancer cell lines exhibited statistically significant decreases in proliferation after KPNA7 knock-down (Fig. 1a, b). In fact, there was a minimal or no difference in Hs700T and T-47D cell numbers between the 72 h and 96 h time points after transfection (Additional file 3: Figure S2A) indicating that KPNA7 silencing leads to an actual growth arrest as cell numbers do not change after 72 h. qRT-PCR was used to confirm the KPNA7 knock-down in all time points (Additional file 3: Figure S2B).

**Fig. 2:**
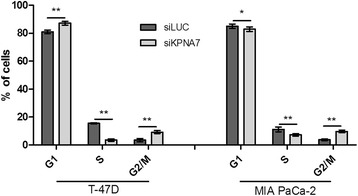
KPNA7 depletion results in reduced number of S-phase cells. The cell cycle distributions were analyzed using flow cytometry 96 h after transfection of siRNAs against KPNA7 or LUC control gene. The experiment was repeated at least twice. The mean and SD of six replicates from a representative experiment are shown.*p < 0.05, **p < 0.01

To confirm the specificity of the observed growth effect, the KPNA7 siRNAs were also transfected into both normal (hTERT-HPNE) and cancerous (MDA-MB-453) cells that completely lack KPNA7 expression (Additional file 2: Figure S1) and no changes in cell growth were observed (Fig. 1c). Since the efficacy of the KPNA7 siRNAs cannot be measured in these cells, parallel transfections with siRNAs against the *SMAD5* gene were performed and led to a 70% reduction in mRNA level, thus indicating that the experimental conditions were appropriate. To evaluate the possible mechanisms behind the growth inhibition phenotype, cell cycle profiles were determined in one breast and one pancreatic cancer cell line (T-47D and MIA PaCa-2, respectively) 96 h after transfection with KPNA7 and LUC control siRNAs. These analyses revealed that KPNA7 inhibition resulted in a diminished fraction of proliferating S-phase cells, with a concomitant increase in G2-phase cells (Fig. 2). The fraction of sub-G1 cells was not altered (data not shown). These results are in agreement with our previous data on Hs700T cells [28].

### Correct assembly of the mitotic spindle is affected by KPNA7 depletion

**Fig. 3:**
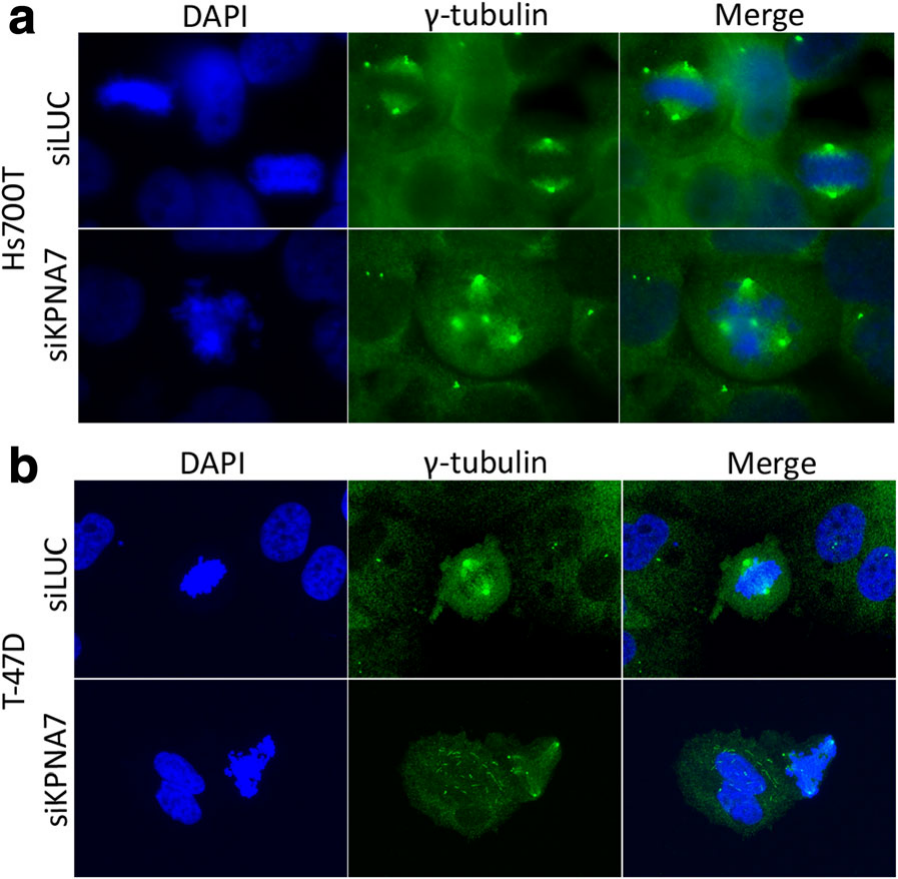
Mitotic KPNA7-silenced cells exhibit aberrant number of centrosomes and an abnormal organization of the mitotic spindle. **a** Hs700T pancreatic cancer cells and (**b**) T-47D breast cancer cells were transfected with either KPNA7 or control LUC siRNA and γ-tubulin (green) was immunofluorescently labeled 96 h later. Nuclei were counterstained with DAPI (blue)

The growth arrest phenotype and altered cell cycle distributions of siKPNA7-treated cells led us to hypothesize that mitosis is disturbed in these cells. To address this issue, we studied the organization of centrosomes by staining their major structural component γ-tubulin, again in one pancreatic and one breast cancer cell line 96 h after transfection with KPNA7 and LUC control siRNAs. Unfortunately, the MIA Paca-2 cells turned out to grow in thick colonies partially on top of each other and had very dense nuclei and thus they were hard to image properly. Therefore, the Hs700T and T-47D cells were used these and all subsequent experiments. The number of centrosomes and the structure of the mitotic spindles was normal, with two centrosomes at the opposite ends of the cell and chromatin aligned correctly in the middle of the nucleus, in 100% and 98% of the mitotic siLUC transfected Hs700T and T-47D control cells, respectively (Fig. 3). In contrast, after KPNA7 silencing, 18% of mitotic Hs700T and 20% of mitotic T-47D cells harbored an abnormal number of centrosomes (Fig. 3). Typically, three to four centrosomes were observed, leading to distortion of the mitotic spindle and subsequent aberrant alignment of the chromatin.

### KPNA7 silencing results in distinct changes in nuclear morphology

**Fig. 4:**
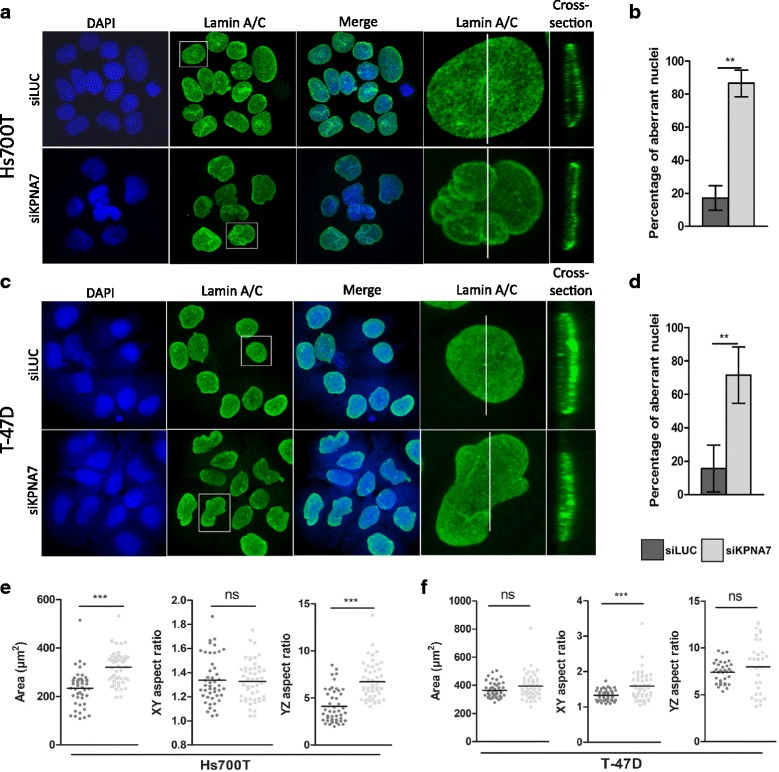
KPNA7 is vital for the maintenance of proper nuclear morphology. **a** Hs700T pancreatic cancer cells and (**c**) T-47D breast cancer cells were treated with control (LUC) or KPNA7 siRNA and stained with lamin A/C antibody (green) 96 h post-transfection. The nuclei were counterstained with DAPI (blue). White squares indicate an individual cell for which an enlarged image is shown and white vertical lines pinpoint the location of which a cross-section of the nucleus is illustrated. The percentages of normal vs. aberrant nuclei were determined for (**b**) Hs700T and (**d**) T-47D cells from a minimum of six microscopic images. The mean and SD of the replicates are shown. **e**, **f** The nuclear area, XY aspect ratio and YZ aspect ratio were determined from 50 nuclei using the ImageJ software. The median is indicated with a horizontal line. **p < 0.01, ***p < 0.005

**Fig. 5:**
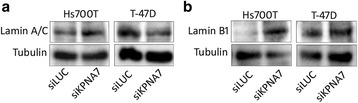
KPNA7 depletion induces alterations in the amounts of lamin proteins. Hs700T and T-47D cells were treated with control or KPNA7 siRNA and Western blotting performed 96 h after transfection with (**a**) lamin A/C or (**b**) lamin B1 antibodies. Tubulin was used as a loading control. The expression levels were quantitated using ImageJ software and were normalized against the tubulin loading control. The figures indicate relative protein expression levels for the siKPNA7 transfected cells as compared to the corresponding siLUC controls

Based on the DAPI-staining in the experiments described above, we noted that the KPNA7-silenced interphase cells had aberrant nuclear shape. To better visualize this effect, the cells were stained for lamin A/C, the major component of the nuclear lamina, revealing distinct lobular nuclear morphology in Hs700T cells (Fig. 4a). The quantitation of the number of normal vs. aberrant nuclei showed a dramatic increase in the fraction of abnormal nuclei from 17% in the control cells to 86% in the KPNA7-silenced cells (*p* < 0.01, Fig. 4b). The analysis of nuclear size and shape showed that the siKPNA7-treated Hs700T cells had significantly larger nuclei than the control cells (*p* < 0.01, Fig. 4f). The aspect ratio (calculated as indicated in Additional file 4: Figure S3 both in XY and YZ dimensions) of the nuclei in XY dimension was not affected, but a significant increase in YZ aspect ratio was observed (*p* < 0.005, Fig. 4f). These data indicate that in Hs700T cells KPNA7 depletion leads to the flattening of the nuclei, which was also visible in the cross-sectional view of the nuclei (Fig. 4a). In T-47D cells, KPNA7 depletion resulted in an elongated, distorted shape of the nuclei (Fig. 4c). Again, the number of aberrant nuclei was significantly increased from 16% in the control cells to over 70% in the KPNA7-silenced cells (*p* < 0.01, Fig. 4d). A small trend towards enlarged nuclear area and YZ aspect ratio was observed while the XY aspect ratio was significantly increased (Fig. 4g), thus providing quantitative confirmation on the elongation of the nuclei. Western blot analysis of the amount of lamin A/C protein showed no major changes in Hs700T cells but a noticeable decrease in T-47D cells after KPNA7 silencing (Fig. 5a).

**Fig. 6:**
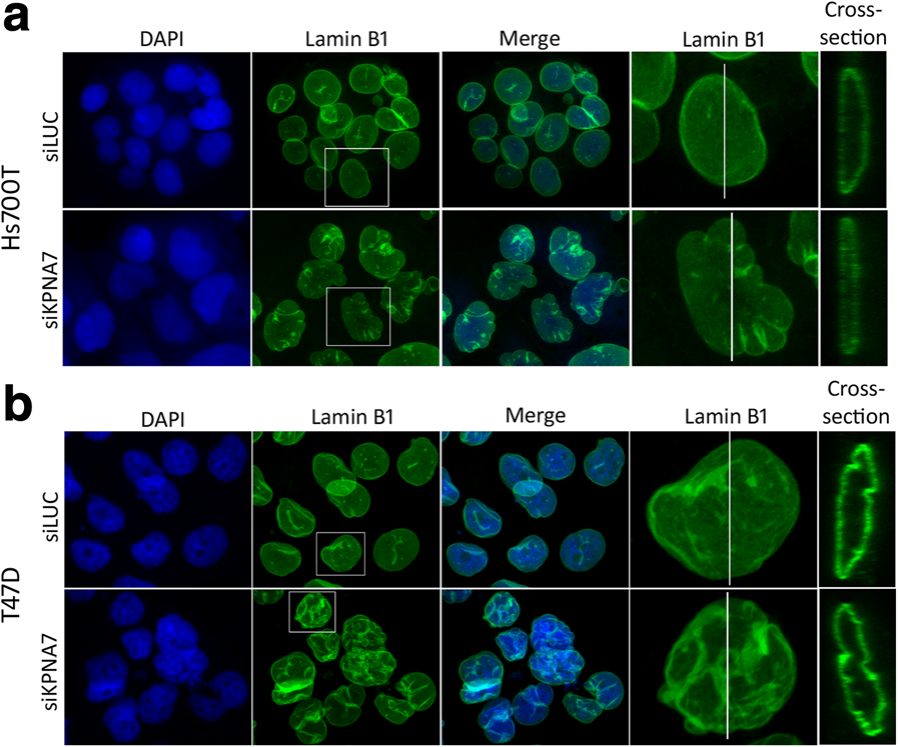
KPNA7 silencing causes rearrangement of lamin proteins. **a** Hs700T and (**b**) T-47D cells were treated with control or KPNA7 siRNA and lamin B1 (green) was immunofluorescently labeled 96 h post-transfection. The nuclei were counterstained with DAPI (blue). The white squares indicate an individual cell for which an enlarged image is shown and the white vertical lines pinpoint the spot for which a cross-section of the nucleus is illustrated

To further assess the impact of KPNA7 depletion on nuclear structure, immunofluorescent staining of lamin B1, another major component of the nuclear lamina, was performed and unveiled radical reorganization and folding of the protein into the lobules of the nuclei of Hs700T and T-47D cells (Fig. 6a and b). The flattening of the Hs700T nuclei in the siKPNA7-treated cells was again clearly visible. Interestingly, Western blotting showed that the amount of lamin B1 protein was clearly increased in the siKPNA7-treated Hs700T cells compared to the corresponding controls whereas no alterations were seen in T-47D cells (Fig. 5b). To evaluate whether the alterations in nuclear shape are caused by the activation of contractility of the actin stress fibers, phosphorylated myosin light chain II (pMLCII) was immunofluorescently labeled but revealed no radical changes between the control and siKPNA7-treated cells in either Hs700T or T-47D cell line (Additional file 5: Figure S4).

Both the A- and B-type lamins have been shown to interact with nucleoporin 153 (NUP153) [30], one of the components of the inner ring of the NPC [31]. Cells depleted of NUP153 exhibit decreased proliferation and G1 arrest of the cell cycle, and NUP153 has also been implicated in the proper organization of nuclear lamina [31]. Immunofluorescent staining of NUP153 was carried out to evaluate whether KPNA7 silencing also influences the number or localization of the NPCs (Additional file 6: Figure S5A,C). Quantitative analysis indicated that the KPNA7 silenced Hs700T cells harbored slightly more NUP153 puncta per 100 μm^2^ than the control cells (Additional file 6: Figure S5B). The distribution of NUP153 spots between the apical and basal sides of the cells was not affected (data not shown). In T-47D, there were no distinct differences in NUP153 staining patterns between the siKPNA7 transfected and the control cells (Additional file 6: Figure S5B). Western blot analysis of the NUP153 protein levels revealed no alterations in the cellular amount of the protein in either cell line (Additional file 6: Figure S5D).

## Discussion

The complex network of proteins comprising the nuclear transport machinery is a critical player in maintaining the proper function of eukaryotic cells. In addition to nuclear transport, these proteins also contribute to other key cellular processes, such as the regulation of cell division [18, 19]. Changes in the functions of the transport proteins, e.g. due to altered expression levels, may thus lead to a variety of cellular defects, with cancer being an ultimate example [9].

In an earlier study, we showed that KPNA7 is an important regulator of pancreatic cancer cell growth in cell lines harboring amplification and high-level overexpression of the gene [28]. Moreover, lower levels of KPNA7 expression was detected in cancer cells without amplification whereas no or very low level expression expression was found in normal adult tissues with the exception of ovary and trachea [28], indicating re-activation of the gene in cancer cells. This led us to question whether low level KPNA7 expression also confers a growth benefit to cancer cells. The present study demonstrates that KPNA7 knock-down consistently decreased cell growth in all cell lines regardless of the endogenous expression level, although the most drastic effect was indeed seen in Hs700T cells with the highest KPNA7 expression. Furthermore, our data demonstrate that KPNA7 depletion results in a genuine growth arrest, as the knock-down cells exhibited minimal or no proliferation between 72 h and 96 h post-transfection. These results suggest that even a low amount of KPNA7 yields a growth advantage to cancer cells. In addition, the growth inhibition phenotype was detected both in pancreatic and breast cancer cell lines, indicating that the role of KPNA7 is not limited to pancreatic cancer. A similar growth regulatory function has been previously established for KPNA2, which is the closest relative of KPNA7 and is known to promote cell proliferation in many malignancies [16, 32–34]. KPNA7 inhibition also led to alterations in the cell cycle, most notably manifesting as decreased fraction of proliferating S-phase cells, thereby partly explaining the growth defects. This finding is in accordance with our previous data that highlighted proteins participating in cell cycle regulation as KPNA7 cargo candidates [35]. It is thus plausible that the growth inhibiting effects of KPNA7 silencing are caused by diminished transport of cell cycle regulators to the nucleus.

The growth-promoting role and the expression pattern of KPNA7 makes it an interesting target for the development of cancer therapies. Inhibitors against KPNB1 and Exportin-1, a nuclear export factor of the karyopherin superfamily, have been tested in different cancers in clinical trials [36]. However, their development has been hindered by toxicities [36], probably due to their ubiquitous expression and essential function in healthy tissues. The targeting of KPNA7 would avoid this problem as its expression is in the current light mainly limited to cancer. On the other hand, the high conservation of the KPNA family members might provide an obstacle for drug development by creating unspecific effects. As Kelley et al. reported, KPNA7 is the most divergent member of the family [23] and might hence represent the most suitable drug target among the KPNAs. In addition, recent computational analysis implicated KPNA7 as a potential biomarker for pancreatic cancer [37].

Immunofluorescent analysis of γ-tubulin revealed an abnormal number of centrosomes and mitotic spindle poles in a notable fraction of the siKPNA7-treated cells, with chromatin being pulled by three or more mitotic spindles towards as many centrosomes. Mislocalization of the GEF Ran guanine nucleotide exchange factor (RCC1), which has a key role in maintaining the appropriate Ran-GTP gradient, is also known to result in multipolar spindles [20]. RCC1 has an NLS and it has been shown to be transported into the nucleus by the KPNA3 and KPNB1 complex [20]. Our previous study pinpointed the GEF Ran-binding protein 10 (RANBP10) as well as multiple other microtubule-associated proteins as putative KPNA7 cargos [35] and thus their diminished transport to the nucleus in KPNA7 silenced cells may contribute to aberrant spindle formation. However, KPNA7 itself has been shown to localize to the spindle structures in murine cells [25], suggesting that it may also directly influence spindle formation. Moreover, KPNB1 was demonstrated to regulate the formation of the spindle via its importin alpha binding (IAB) domain, further supporting the possible role of karyopherins in the regulation of spindle formation [38].

KPNA7 depletion also induced distinct changes in the nuclear morphology in both Hs700T pancreatic and T-47D breast cancer cells. The nuclear lobulation does not seem to be fatal for the cells, but may explain the growth arrest observed. Changes in nuclear shape are usually attributed to different lamin proteins, i.e. intermediate filaments that form the nuclear lamina scaffold adjacent to the inner nuclear membrane [39]. For example, mutations in lamin proteins have been linked to many diseases known as laminopathies, which are associated with altered nuclear structure and shape [40]. Lobulated nuclei, similar to those in KPNA7 depleted Hs700T cells, are seen in the Hutchinson–Gilford progeria syndrome (HGPS), which is caused by mutations in lamin A [41]. However, reduction in total amount of lamin B1 has been reported in HGPS [40] whereas we observed an increase in lamin B1 protein. Together these data indicate that the mechanisms behind the lobulation of nuclei are diverse and different alterations in lamin proteins contribute to this phenomenon.

Lamins A and C confer stiffness to the nuclei, stabilizing the nucleus against stress, whereas B-type lamins lend elasticity [42, 43]. The increased amount of lamin B1 in KPNA7 silenced Hs700T cells is thus likely to render the nuclear lamina more elastic. This point is supported by the increased nuclear area, the changed YZ aspect ratio of the nuclei, and the cross-sectional views of the lamin protein stainings, all suggesting that the Hs700T nuclei are flattened after the siKPNA7 treatment. In T-47D cells, no increase in lamin B1 was detected but lamin A/C amount was decreased. This might have a similar impact on nuclear rigidity as lamin B1 increase, since lamin A/C depleted cells show reduced nuclear stiffness [43, 44]. The nuclei of the T-47D cells are relatively flat to begin with, as evidenced by high YZ aspect ratios, possibly explaining why KPNA7 silencing did not induce similar flattening of the nuclei as seen in Hs700T but instead resulted in elongated, elliptical nuclear shape.

Loss of lamin B1 levels have been associated with cellular senescence, a potent tumor-suppressive mechanism that leads to an irreversible cell cycle exit, and the lamin B1 loss has also been suggested as a senescence-associated biomarker [45, 46]. For example, in WI-38 human lung embryonic fibroblast cells, silencing of lamin B1 induced premature senescence and, vice versa, the overexpression delayed the onset of senescence [47]. However, in other studies upregulation of lamin B1 has been linked with induction of senescence [47]. The current conclusion thus seems to be that the change in lamin B1 levels is not fully responsible for the senescence phenotype [46, 48]. This notion is in concert with our previous data [28] showing that the siKPNA7-treated Hs700T cells, despite the altered lamin B1 amount demonstrated here, do not exhibit senescence-like characteristics.

It is interesting that the majority (80%) of the siKPNA7-treated cells exhibited changes in nuclear morphology, whereas only 20% of the mitotic cells presented with aberrant multipolar spindles. Based on these observations one could conclude that the main impact of KPNA7 depletion is on lamins and nuclear morphology. Nuclear lamins have been shown to play a role in the formation of the mitotic spindle matrix [49], and hence the observed aberrant mitosis may be related to abnormal spindle formation due to the improper function of the lamins. However, our study does not provide solid evidence on the chronological sequence of events between the mitotic defects and altered nuclear morphology, or whether these phenomena are indeed mechanistically connected.

## Conclusions

In this study, we demonstrate that the silencing of KPNA7, the least studied member of the karyopherin alpha family of nuclear transport proteins, leads to a distinct inhibition of pancreatic and breast cancer cell proliferation, in spite of the endogenous expression level. Our data also demonstrate that KPNA7 has a critical role in the regulation of mitosis through the proper organization of the mitotic spindle and acts in the maintenance of the nuclear envelope structure and nuclear morphology. These results shed new light on the function of KPNA7 in the regulation of cancer cell growth and the maintenance of nuclear envelope environment, and thereby further enhance our knowledge on the role of nuclear transfer proteins in cancer pathogenesis.

## Additional files

Additional file 1: **Table S1.** Sequences of the qPCR primers used in this study. **Table S2.** Cell numbers per well used in this study for cell proliferation, cell cycle and immunofluorescent assays. **Table S3.** KPNA7 silencing efficiencies of the cell lines used in Fig. 1 24 h after siRNA treatment. (DOCX 17 kb)

Additional file 2: **Figure S1.** KPNA7 mRNA expression levels were determined in the indicated cancer or normal cell lines by qRT-PCR. The expression values were normalized against a housekeeping gene HRPT. The data on the pancreatic cancer cell lines are in accordance with what was previously reported [28], and are shown here to allow comparison between all cell lines used in this study. (PDF 342 kb)

Additional file 3: **Figure S2.** KPNA7 knock-down induces a growth arrest phenotype in pancreatic and breast cancer cells. (A) Hs700T and T-47D cells were transfected with KPNA7 or control siRNAs and the cell numbers were counted 72 h and 96 h post-transfection. (B) KPNA7 expression levels were determined with qRT-PCR at 24, 48, 72 and 96 h after transfection to confirm the level of knock-down. (PDF 725 kb)

Additional file 4: **Figure S3.** Schematic representation of the calculation of the aspect ratio both in XY and YZ directions. (PDF 3613 kb)

Additional file 5: **Figure S4.** KPNA7-silencing does not lead to the formation of stress fibers. (A) Hs700T and (B) T-47D cells were transfected with KPNA7 or control siRNAs and phospho-Myosin light chain 2 (pMLCII) IF staining (green) performed 96 h after transfection. The nuclei were counterstained with DAPI (blue) and F-actin with Phalloidin (red). (PDF 2768 kb)

Additional file 6: **Figure S5.** KPNA7 depletion does not have a major impact on NPCs. Hs700T (A) and T-47D (C) cells were transfected with KPNA7 or control siRNAs and NUP153 IF staining (green) performed 96 h after transfection. The nuclei were counterstained with DAPI (blue). The white squares indicate an individual cell for which an enlarged image is shown and the white vertical lines pinpoint the location for which a cross-section of the nucleus is illustrated. (C) NUP153 spots were counted with ImageJ software from 100 μm^2^ area. The mean and SD of 6 nuclei are shown. (D) Western blotting of NUP153 was performed 96 h after siRNA transfection. Tubulin was used as a loading control. (PDF 1538 kb)

## Abbreviations

DAPI: 4′,6-diamidino-2-phenylindole
GAP: GTPase activating protein
GEF: Guanine nucleotide exchange factor
HGPS: Hutchinson–Gilford progeria syndrome
IAB: Importin alpha binding domain
KPNA (2, 4, 7,): Karyopherin alpha (2, 4, 7)
KPNB1: Karyopherin beta 1
LUC: Firefly luciferase
NLS: Nuclear localization signal
NPC: Nuclear pore complex
NUP153: Nucleoporin 153
p53: Tumor suppressor protein 53
pMLCII: Phosphorylated myosin light chain II
RANBP10: Ran-binding protein 10
Ran-GTP: GTP-binding nuclear protein Ran
RCC1: Ran guanine nucleotide exchange factor
SAF: Spindle assembly factor
siRNA: small interfering RNA
