# Correction to: Depletion of nuclear import protein karyopherin alpha 7 (KPNA7) induces mitotic defects and deformation of nuclei in cancer cells

**DOI:** 10.1186/s12885-018-5234-4

**Published:** 2019-01-14

**Authors:** Elisa M. Vuorinen, Nina K. Rajala, Teemu O. Ihalainen, Anne Kallioniemi

**Affiliations:** 1 BioMediTech Institute and Faculty of Medicine and Life Sciences, University of Tampere, 100, 33014 Tampere, PL Finland; 2 BioMediTech Institute and Faculty of Biomedical Sciences and Engineering, Tampere University of Technology, University of Tampere, 100, 33014 Tampere, PL Finland; 3 Tampere Imaging Facility, BioMediTech Institute and Faculty of Medicine and Life Sciences, University of Tampere, 100, 33014 Tampere, PL Finland; 4 Fimlab Laboratories, Biokatu 4, 33520 Tampere, Finland

**Correction to: BMC Cancer (2018) 18:325**

**https://doi.org/10.1186/s12885-018-4261-5**

Following publication of the original article [1], the authors notified us that the Additional file 1 contains reviewer comments instead of the Supplementary tables. Supplementary tables are now provided in the Addtional file 1:

## Additional file

Additional file 1: **Table S1.** Sequences of the qPCR primers used in this study. **Table S2.** Cell numbers per well used in this study for cell proliferation, cell cycle and immunofluorescent assays. **Table S3.** KPNA7 silencing efficiencies of the cell lines used in Fig. 1 24 h after siRNA treatment. (DOCX 17 kb)
