# Supplementary figures and images for: Depletion of nuclear import protein karyopherin alpha 7 (KPNA7) induces mitotic defects and deformation of nuclei in cancer cells
Source: BMC Cancer. 2018 Mar 27;18:325. doi: 10.1186/s12885-018-4261-5 (PMC5870926; doi:10.1186/s12885-018-4261-5)

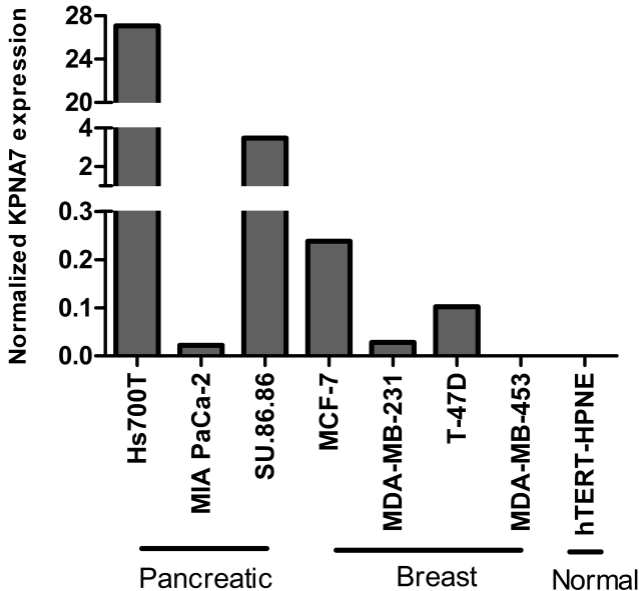

Supplement: Supplementary file 2 — Figure S1. KPNA7 mRNA expression levels were determined in the indicated cancer or normal cell lines by qRT-PCR. The expression values were normalized against a housekeeping gene HRPT. The data on the pancreatic cancer cell lines are in accordance with what was previously reported [28], and are shown here to allow comparison between all cell lines used in this study. (PDF 342 kb) [file 12885_2018_4261_MOESM2_ESM.pdf]

A

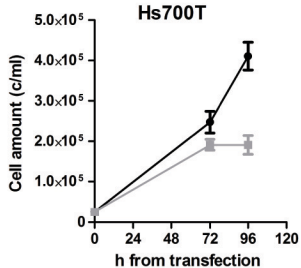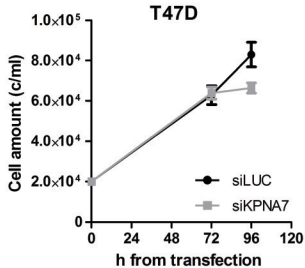

B

| Cell line     | Silencing efficiency (%) |     |     |     |
|---------------|--------------------------|-----|-----|-----|
|               | 24h                      | 48h | 72h | 96h |
| <b>Hs700T</b> | 86                       | 89  | 85  | 76  |
| <b>T-47D</b>  | 87                       | 78  | 60  | 45  |

Supplement: Supplementary file 3 — Figure S2. KPNA7 knock-down induces a growth arrest phenotype in pancreatic and breast cancer cells. (A) Hs700T and T-47D cells were transfected with KPNA7 or control siRNAs and the cell numbers were counted 72 h and 96 h post-transfection. (B) KPNA7 expression levels were determined with qRT-PCR at 24, 48, 72 and 96 h after transfection to confirm the level of knock-down. (PDF 725 kb) [file 12885_2018_4261_MOESM3_ESM.pdf]

$$AR_{xy} = \frac{\Delta x}{\Delta y}$$

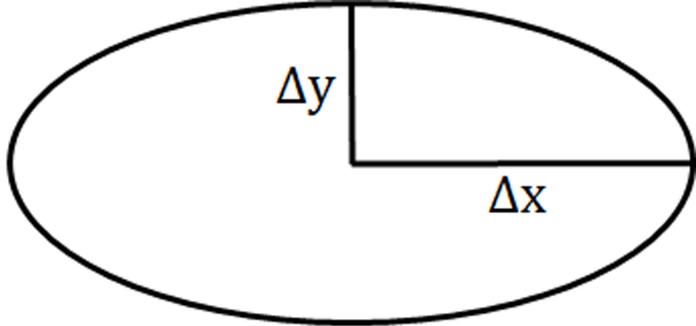

$$AR_{yz} = \frac{\Delta y}{\Delta z}$$

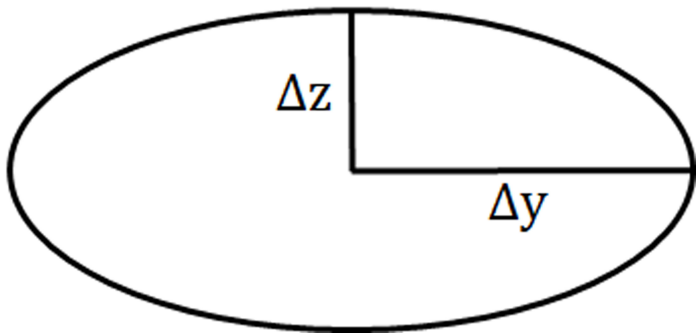

Supplement: Supplementary file 4 — Figure S3. Schematic representation of the calculation of the aspect ratio both in XY and YZ directions. (PDF 3613 kb) [file 12885_2018_4261_MOESM4_ESM.pdf]

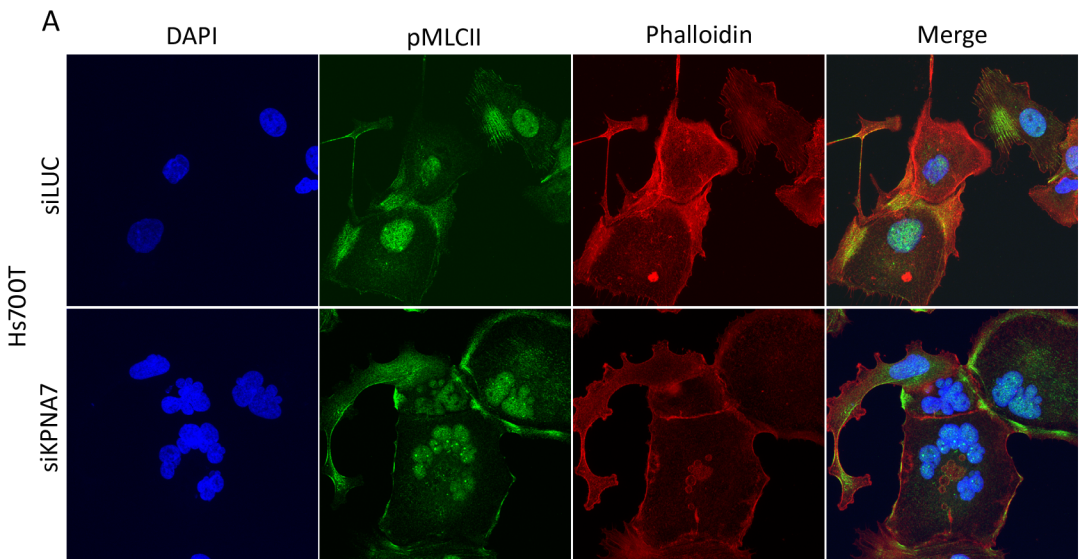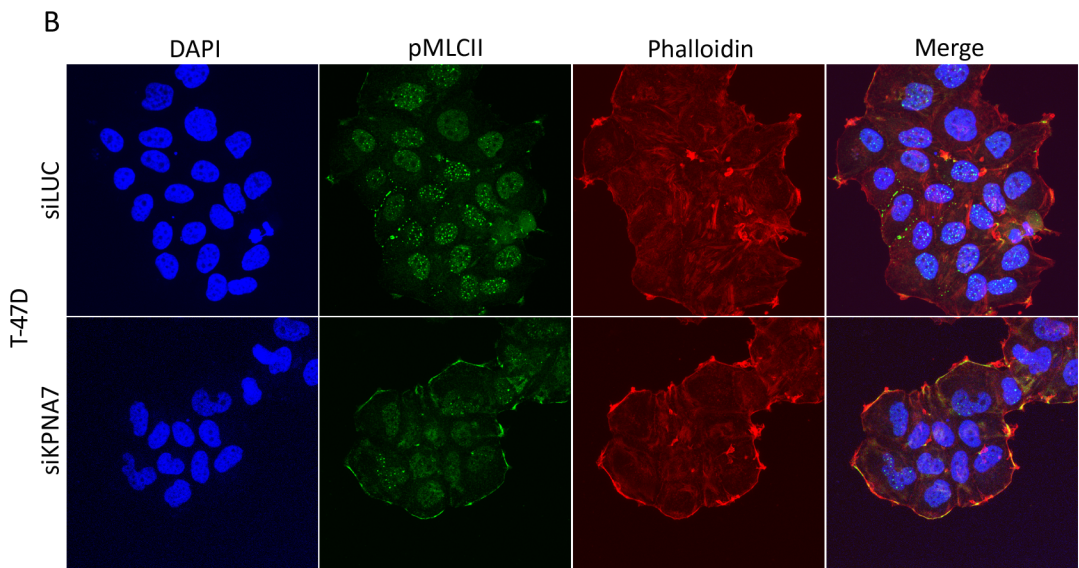

Supplement: Supplementary file 5 — Figure S4. KPNA7-silencing does not lead to the formation of stress fibers. (A) Hs700T and (B) T-47D cells were transfected with KPNA7 or control siRNAs and phospho-Myosin light chain 2 (pMLCII) IF staining (green) performed 96 h after transfection. The nuclei were counterstained with DAPI (blue) and F-actin with Phalloidin (red). (PDF 2768 kb) [file 12885_2018_4261_MOESM5_ESM.pdf]

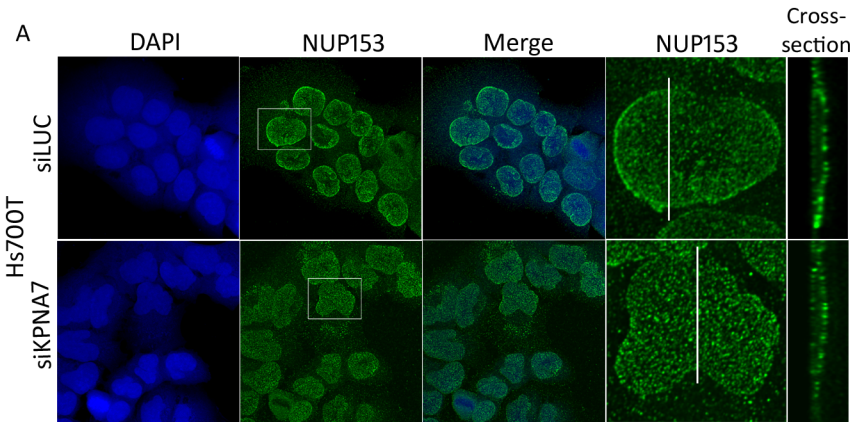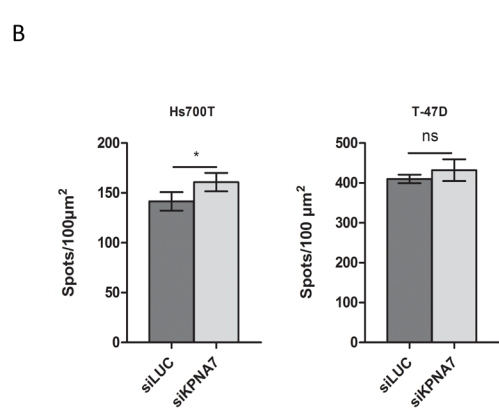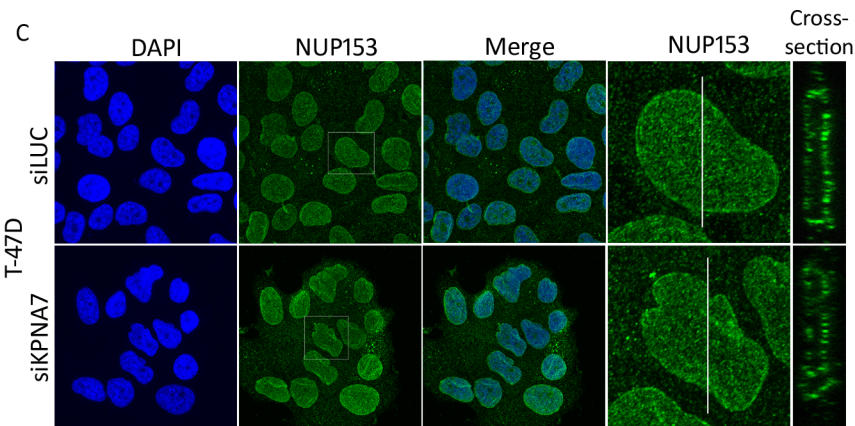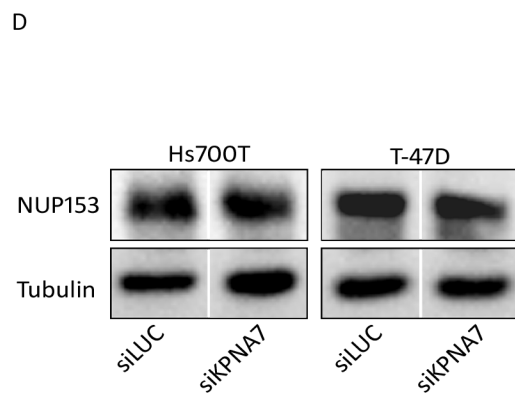

Supplement: Supplementary file 6 — Figure S5. KPNA7 depletion does not have a major impact on NPCs. Hs700T (A) and T-47D (C) cells were transfected with KPNA7 or control siRNAs and NUP153 IF staining (green) performed 96 h after transfection. The nuclei were counterstained with DAPI (blue). The white squares indicate an individual cell for which an enlarged image is shown and the white vertical lines pinpoint the location for which a cross-section of the nucleus is illustrated. (C) NUP153 spots were counted with ImageJ software from 100 μm2 area. The mean and SD of 6 nuclei are shown. (D) Western blotting of NUP153 was performed 96 h after siRNA transfection. Tubulin was used as a loading control. (PDF 1538 kb) [file 12885_2018_4261_MOESM6_ESM.pdf]
