# Supplementary material for: Depletion of nuclear import protein karyopherin alpha 7 (KPNA7) induces mitotic defects and deformation of nuclei in cancer cells
Source: BMC Cancer. 2018 Mar 27;18:325. doi: 10.1186/s12885-018-4261-5 (PMC5870926; doi:10.1186/s12885-018-4261-5)
Supplement: Supplementary file 1 — Table S1. Sequences of the qPCR primers used in this study. Table S2. Cell numbers per well used in this study for cell proliferation, cell cycle and immunofluorescent assays. Table S3. KPNA7 silencing efficiencies of the cell lines used in Fig. 1 24 h after siRNA treatment. (DOCX 17 kb) [file 12885_2018_4261_MOESM1_ESM.docx]

**Response to review comments**

We would like to thank both of the reviewers for their time and effort in reviewing our manuscript. We appreciate the constructive feedback and the chance to improve our paper. We hope that the revisions made to the manuscript have strengthened the arguments presented. Please find below our detailed point-by-point responses to the comments provided.

**Reviewer # 1:**

***Comment 1.*** *In the introduction, the authors refer to KPNA2 overexpression in lesions, and imply other KPNAs have similar functions in cancer. But in the study, one of the breast cancer cell lines (MDA-MB-453) had no KPNA7 expression. It indicates that KPNA7 overexpression could be only found in some cancers, so it may not be a consistent cancer biomarker. In addition, loss function of KPNA7 in this cancer cell line may suggest that the role of KPNA7 is dispensable. To test this, the authors should examine the cell proliferation by knocking in KPNA7 in* *MDA-MB-453 cell lines. The text should be revised according to these points.*

**Response.** We did not claim that KPNA7 is a consistent cancer biomarker although it indeed appears to be reactivated in a substantial portion of pancreatic and breast cancer cell lines. In the case of MDA-MB-453 cells, reactivation of KPNA7 expression does not occur for an unknown reason. However, one cannot argue that KPNA7 expression is lost in this cell line because the gene is not expressed in normal breast tissue either. The analysis of KPNA7 dispensability by knock-in experiments is thus beyond the scope of this study. Finally, we want to mention a very recent publication (published after the initial submission of our paper) that identified KPNA7 as a highly potential pancreatic cancer biomarker. This information has now been included in the discussion (page 14).

***Comment 2.*** *The authors started with several cell lines for cell proliferation assays, but the assays from Fig 2 to 6 were only for T-47D, MIA PaCa-2 or Hs700T. The authors need to state the rationales for the selection.*

**Response.** Our previous paper (Laurila et al. 2014 Exp Cell Res) described the effect of KPNA7 silencing in two high KPNA7-expressing pancreatic cancer cell lines, Hs700T and AsPC-1. Here, we extended these results to breast cancer cell lines as well as to additional pancreatic cancer cell lines with different endogenous KPNA7 expression levels and showed that in all of them, KPNA7 silencing leads to dramatic growth inhibition. One breast (T-47D) and one pancreatic (MIA Paca-2) cancer cell line were then selected for the remaining experiments. Unfortunately, the MIA Paca-2 cells turned out to grow in thick colonies partially on top of each other and had very dense nuclei and thus they were hard to image properly. Therefore, we replaced it with Hs700T cells in the nuclear morphology experiments. The cell cycle data on KPNA7 depleted Hs700T cells has been already presented in our previous paper (Laurila et al. 2014) and were thus not included in here to avoid duplicate publication. The rationales for the cell line choices have now been added to the manuscript (pages 9 and 10).

***Comment 3.*** *The comparison in Fig 2 was not clear. Why is the cell # at G2/M phase of siKPNA7 higher than the control? Same to G1 phase. Provide more discussion about the data.*

**Response.** In terms of cell cycle analyses, one must bear in mind that the sum of all the cell cycle fractions is 100%. When one fraction (in our case the S-fraction) is diminished, it inevitably results in increase in the other fractions and thus we feel that further discussion of this fact is not relevant.

***Comment 4.*** *Fig 4E was not referred to in the text. In addition, the notation in Fig 4E was not defined and should be consistent with the following statement (i.e., X, Y, Z). The authors may consider removing Fig 4E from the manuscript.*

**Response.** Fig. 4E has been moved to supplements as new Supplementary Figure S3 and is referred to in the Results section. The notations have been changed to correspond to the dimensions used in the text (X, Y, Z).

***Comment 5.*** *The statistical analysis throughout the results needs to be validated. For example, in Fig 1B, the p value of MCF-7 was noted being smaller than the p value of MDA-MB-231. But the SD of MDA-MB-231 was less than the SD of MCF-7. Same concerns for Fig 4F and G. Due to the overlapping error bar, could it be possible that p<0.005?*

**Response.** A statistician was consulted and all statistical analyses were re-checked and found to be correct. The plots in question depict the mean and SD of the data. Since the data is not normally distributed, the statistical difference between the groups was assessed using the Mann-Whitney test, which compares the medians of the groups. Due to few divergent data points (especially in Fig. 4), the SDs are quite large, but the statistical significances are indeed as indicated in the original submission. In Fig. 4, the n-value is quite big in both groups (50), which makes the results significant even though the SDs overlap. To better illustrate the data in this figure, the histograms have been replaced with dot plots.

***Comment 6.*** *The last paragraph on p14 was discussing KPNA7 depletion in nuclear morphology. However, Fig 5 shows Hs700T with siKPNA7 had no influence on Lamin A/C, so how is KPNA7 depletion related to lobulated nuclei caused by mutations in Lamin A? KPNA7 depletion was seen from T-47D cells but different nuclear morphology was observed.*

**Response.** Perhaps a minor misunderstanding has taken place. We do not postulate that KPNA7 depletion is related to lobulated nuclei caused by mutations in Lamin A/C but simply wanted to provide an example of known alterations of Lamin A/C in disease (in this case HGSP). We have now clarified this issue and state that the mechanisms behind the lobulation of nuclei are diverse and different alterations in lamin proteins contribute to this phenomenon (page 15).

***Comment 7.*** *Is KPNA7 a druggable target? This information should be included in the implication of discussion.*

**Response.** It is indeed possible that KPNA7 could act as a drug target. A chapter discussing the druggability of KPNA7 has been added to the discussion (page 14).

***Comment 8.*** *Correct grammar errors and typos (e.g., p13 line 22).*

**Response.** The manuscript has been proofread and all detected typos and grammar errors have been corrected.

**Reviewer # 2:**

***Comment 1.*** *Could the authors confirm siKPNA7 knockdown in the cell lines they used in the current study (Figure 1)?*

**Response.** The knock-down efficiencies have been added to supplementary material as Supplementary Table S3.

***Comment 2.*** *Could the authors confirm KPNA7 knockdown after 24, 48, 72 and 96 hours post transfection in Supplementary Figure 2 (by Western blotting or RT-qPCR)?*

**Response.** The knock-down efficiencies have been confirmed with qRT-PCR at the requested time points and added to Supplementary Figure S2 as panel B.

***Comment 3.*** *It would be very beneficial if the authors provide quantifications of the western blotting images in Figure 5A, B (bar graphs with statistics).*

**Response.** Quantification of the Western blotting data has been added to Fig. 5 as requested.
