# Supplementary material for: Correction to: Depletion of nuclear import protein karyopherin alpha 7 (KPNA7) induces mitotic defects and deformation of nuclei in cancer cells
Source: BMC Cancer. 2019 Jan 14;19:57. doi: 10.1186/s12885-018-5234-4 (PMC6330744; doi:10.1186/s12885-018-5234-4)
Supplement: Supplementary file 1 — Table S1. Sequences of the qPCR primers used in this study. Table S2. Cell numbers per well used in this study for cell proliferation, cell cycle and immunofluorescent assays. Table S3. KPNA7 silencing efficiencies of the cell lines used in Fig. 1 24 h after siRNA treatment. (DOCX 17 kb) [file 12885_2018_5234_MOESM1_ESM.docx]

Table S1: Sequences of the qPCR primers used in this study

| Gene | Forward primer | | Reverse primer |  | Probe # | |
| --- | --- | --- | --- | --- | --- | --- |
| KPNA7 | cagatccagtcctatgtttcca | cgcttcaatgaccagtttca | |  | | 72 |
| SMAD5 | ggaccaggaagtccatttca | ctgggaattatcttgacccatc | |  | | 51 |

Table S2: Cell numbers per well used in this study for cell proliferation, cell cycle and immunofluorescent assays.

| Cell line | 24-well plate  cells/well | 6-well plate  cells/well |
| --- | --- | --- |
| Hs700T | 25,000 |  |
| MIA PaCa-2 | 20,000 | 75,000 |
| SU.86.86 | 15,000 |  |
| MCF-7 | 30,000 |  |
| MDA-MB-231 | 25,000 |  |
| T-47D | 25,000 | 125,000 |
| MDA-MB-453 | 20,000 |  |
| hTERT-HPNE | 8,000 |  |

Table S3: KPNA7 silencing efficiencies

of the cell lines used in Fig.1 24h after siRNA treatment.

| Cell line | Silencing efficiency (%) |
| --- | --- |
| Hs700T | 84 |
| MIA PaCa-2 | 82 |
| SU.86.86 | >90* |
| MCF-7 | 83 |
| MDA-MB-231 | >90* |
| T-47D | >90* |

*****In these cell lines, the expression levels dropped

below reliable detection limit after knock-down

in the experiment in question.
